# A system for de-identifying medical message board text

**DOI:** 10.1186/1471-2105-12-S3-S2

**Published:** 2011-06-09

**Authors:** Adrian Benton, Shawndra Hill, Lyle Ungar, Annie Chung, Charles Leonard, Cristin Freeman, John H Holmes

**Affiliations:** 1 University of Pennsylvania School of Medicine, Philadelphia, PA; 2 University of Pennsylvania, The Wharton School, Philadelphia, PA; 3 University of Pennsylvania School of Engineering and Applied Science, Philadelphia, PA

## Abstract

There are millions of public posts to medical message boards by users seeking support and information on a wide range of medical conditions. It has been shown that these posts can be used to gain a greater understanding of patients’ experiences and concerns. As investigators continue to explore large corpora of medical discussion board data for research purposes, protecting the privacy of the members of these online communities becomes an important challenge that needs to be met. Extant entity recognition methods used for more structured text are not sufficient because message posts present additional challenges: the posts contain many typographical errors, larger variety of possible names, terms and abbreviations specific to Internet posts or a particular message board, and mentions of the authors’ personal lives. The main contribution of this paper is a system to de-identify the authors of message board posts automatically, taking into account the aforementioned challenges. We demonstrate our system on two different message board corpora, one on breast cancer and another on arthritis. We show that our approach significantly outperforms other publicly available named entity recognition and de-identification systems, which have been tuned for more structured text like operative reports, pathology reports, discharge summaries, or newswire.

## Introduction

Medical message boards (MMBs) serve as forums for emotional support and information exchange, usually for patients with similar conditions. Users of MMBs communicate by asynchronously posting messages to the board in *threads*, groups of messages that are typically centered on a single topic. Because of the sheer number, inexpensiveness, and candid nature of messages posted on these boards, many researchers have begun to treat MMB threads as “virtual focus groups” to gain more knowledge about patient experiences [1-3]. Additionally, our group is currently using MMBs as a source for identifying undocumented adverse effects from drugs and dietary supplements.

As more patients gain access to the Internet and join these communities, more MMB text on patient experiences will become available, providing researchers with further opportunities to investigate. However, in order to adhere to ethical requirements in quoting from or performing research on MMB corpora, all information that may identify the user should be removed. In fact, the University of Pennsylvania’s institutional review board requires this. This information includes personal and usernames, email and postal addresses, telephone numbers, and uniform resource locators (URLs), collectively defined here as *identifiers*. There has been considerable research in the domain of Named Entity Recognition (NER), the task of identifying instances of a particular type, such as people or companies within free text. Many NER systems have been developed and perform reasonably well [4]. However, since MMB text is much more unstructured and noisy than the text for which most NER systems are developed [5], these methods are not as effective at capturing identifiers within MMB posts. For example, running the Stanford Named Entity Recognizer [6] trained on a corpus of US and UK newswires to identify proper names within a random sample of 500 posts resulted in correctly identifying 81.2% of proper names with a precision of 61.7%. This does not take into account any usernames that were present in these documents. In comparison, this same system was originally reported to be able to identify proper names with an F-score of 92.3% over a sample of newswire [6]. We frame the task of de-identifying MMB text as a specialized form of NER.

There is also a well-developed body of research regarding medical document de-identification. Many systems have already been developed to de-identify different types of free text medical documents such as pathology reports, nursing notes, and discharge summaries. Many of these systems rely heavily on heuristics and pattern matching in order to remove identifying information [7-9]. Others have used statistical models in order to detect identifying information, including maximum entropy classifiers [10], support vector machines [11], and conditional random fields (CRFs) [12]. The problem of de-identifying medical records has been addressed by numerous researchers up to this point and performance of some of these systems is exceptional, with F-scores over 98% for the best systems [13]. Unfortunately, these methods that have been tuned to the more regular text of medical documents will not translate easily to de-identifying MMB text. This is because MMB text is extremely noisy with frequent typographical errors, a large variation in possible names, terms and abbreviations that are only specific to Internet posts or even a particular message board (e.g., bilat mx, onc), and the authors frequently refer to their friends and family members in their posts.

In this paper, we present a system that is able to remove phone numbers, e-mail addresses, user resource locators (URLs), proper names, and usernames from MMB text. We focus here specifically on name de-identification.

## Methods

**Figure 1:**
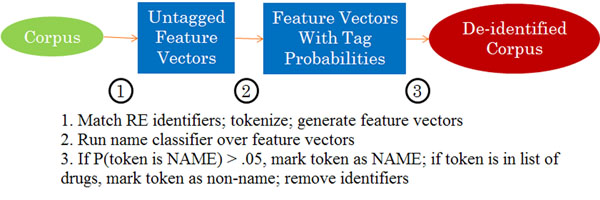
De-identification process

Our system first identifies email addresses, phone numbers, and URLs using regular expressions and tokenizes the rest of the document around these identifiers. It then generates a feature vector for each token. A name classifier is then used to generate tag probabilities for each of these tokens based on its associated vector. All tokens with probability of being a name greater than 0.05 are tagged as names. The token class probability estimate threshold of 0.05 was learned on a development set. On the final pass, all tokens that are tagged as names but names of drugs are untagged. Tokens that had been tagged as identifiers are then removed from the document, replaced with placeholders, and written to a de-identified file. This process is depicted in Fig. 1.

### Corpora

We used two corpora to train and validate our system. The first corpus, the breast cancer (BC) corpus, was generated by downloading the messages from 12 different BC message board sites. Downloaded messages were then cleaned with scripts specifically tailored to the layout of each message board, to fit to a standard format.

The BC corpus contained over 1.2 million messages and 74,000 threads, the majority of which came from http://breastcancer.org or http://komen.org.

In addition to the BC corpus, we also compiled a corpus of arthritis posts gathered from three different arthritis message boards: http://www.healthboards.com/, http://arthritisinsight.com/forum/, and http://boards.webmd.com. We randomly sampled messages from this corpus to generate the test set for validating our system. This corpus was generated in the same manner as the BC corpus and contained over 100,000 messages and 14,000 threads. We selected the test set from this corpus in order to realistically determine how well our system would perform over a completely novel MMB, with different conditions and usernames than the training set.

### Pre-processing

Before names were identified, the corpus was passed through a pre-processing step corresponding to step 1 in Fig. 1. In this step, e-mail addresses, URLs, and phone numbers were identified via regular expressions. For example, e-mail addresses were identified with the regular expression “[\w.]+@\w+(.[\w])*” and phone numbers were identified with “(\d\d\d[-._ ]?)+\d\d\d\d” where *\d* refers to the set of digits and *\w* refers to the set of alphanumeric characters and underscore.

After these identifiers were discovered, the remaining text was split into tokens by whitespace and any punctuation marks. Once the text had been tokenized, our system generated a feature vector for each item in the output. Each token’s feature vector is a set of properties that describe that particular instance of the token, and are used by the name classifier to determine the likelihood that a given token is a name.

### Feature vectors

The features that we used to train the CRF can be grouped into two classes: features that do not rely on the structure of MMBs and those that take advantage of the way that MMBs are structured.

#### MMB non-structure features

The MMB non-structure features tend to be features that would be helpful in identifying names in many different media, not necessarily MMBs. The features that we used to describe each token include the token itself, the token lower-cased, and its length. The case of the token was encoded as either lower, upper, title, or mixed case, each being a binary feature. The token’s two and three character suffixes and prefixes were also included. These features are helpful in identifying names in many different media, since names tend to be capitalized (although to a lesser extent in MMB text) and certain prefixes and suffixes may also indicate a name. We also included the distance of the token (in number of tokens) from the beginning and end of the message as a feature to take advantage of the fact that MMB posts often begin by addressing another user and end with the author’s name.

Membership in each word list was included as a binary feature. We also included features for possible membership in a particular word list. A token was considered a possible member of a word list if it was a distance of one or two edits from a token in that word list. Edit distance from one token to another was defined as the Damerau-Levenshtein distance, the number of additions, substitutions, removals, and transpositions of characters required to transform that token into the other. This feature was useful in identifying tokens that could be misspelled names, even if that particular token was not in any of the system’s word lists. The word lists used to generate these features and sources for each are listed in Table 1. The word lists used are not specific to the domain of MMB text, for the most part. The username word lists are generated by extracting the text in the author field from each message post. The only lists that may be domain specific are the medical term and drug word lists, which were specific to neither breast cancer nor arthritis message board posts.

**Table 1:** Word lists used to generate features

Dictionary	Source
Proper name	Natural language toolkit [18] and Deid system [9,16]
Common word	Ispell, GNU spell-checker dictionary; inspired by Thomas et al. [8]
Stop word	Generic list of very common English words
Medical word	Deid system lexicon [9,16]
Drug word	Generated from the Cerner Multum Drug Lexicon, (Denver, CO)
Honorific	Compiled by hand (e.g., mr., mrs, dr.)
All user	Users that have posted to this message board, generated from “author” field of each message
User variant	Users who have posted to this particular thread, with variants of these names derived automatically (strip digits, split by delimiters/camel case/known names and words)

The vector also includes the features of the two previous tokens and the two next tokens. We included the features of the two previous and following tokens in each feature vector because the system performed better than the conditions where we included no surrounding tokens, included the immediately surrounding tokens, or included three tokens on either side. This may be due to the fact that certain words strongly indicate a proper name (e.g., honorifics).

#### MMB structure features

An MMB corpus can be segmented in several different ways. For example, one can consider each message as a separate document. Likewise, one can consider each thread, all threads within a particular message board, or all messages posted by a particular user as separate documents. Certain words will be repeated much more frequently within a document than in the entire corpus. In other words, they are “document-specific”. Many of these document-specific words are in fact names. At the level of a thread, these are most likely the names of the users participating in that thread. At the level of a particular user, they would likely be their own name and other users that they frequently converse with. We use the term frequency-inverse document frequency metric (tf-idf) in two ways: by treating all messages that belong to a particular message board as a document, and by treating all messages that a particular user posts as a document. Each token in the document is ranked by its tf-idf value and its rank is included in the feature vector (e.g., inTop1%=TRUE).

Tf-idf is defined as follows:(1)(2)

where *tf_i_*_,_*_j_* is the frequency of a particular token *i* within a document *j* normalized by the square-root of the total number of tokens within *j*, *N* is the total number of documents within the corpus, and *n_i_* is the total number of documents that contain at least one instance of token *i*. This metric favors terms that occur many times within the current document, but occur in very few other documents. The list below shows the top 25 tokens when ranking by tf-idf in the http://breastcancer.org message board, which is one of 12 different message board sites that comprise the BC corpus.

-- Likely names (obvious name or variant in username dictionary): mjb, shirlann, mena, nicki, barbe, marsha, ravdeb, lisa, shokk, hippie96321, laura, odalys, jankay, harley, kaygirl, janiemarie, ginadcnj, spar, vickie, binney, luann

-- Not likely to be names: hugs, tx, onc, dh

Another virtue of this type of metric is that it tends to assign higher values to names that are rarer in general and may not occur in the proper name or even username lists. A similar feature that takes advantage of the fact that a particular name will occur multiple times in a particular document but not throughout the corpus was used by Minkov, Wang, and Cohen to identify names in e-mail messages [14].

We also used the likelihood that a token would appear near the beginning or end of a paragraph over the entire corpus scaled by the logarithm of the number of times that token appeared in the corpus as a feature. This was used to take advantage of the fact that although a particular name may not be used to end a message or greet another user at its current location, perhaps it has been frequently used in this manner in other messages.

Table 2 provides an overview of the feature set that our system uses and lists examples of each feature type.

**Table 2:** Overview of features used by our system

***Feature***	***Example***
**MMB non-structure features**	
token	Kathy
token lower-cased	kathy
length	5
case	isLower=True, isCapitalized=False, …
suffix/prefix	suffix2=hy, prefix2=ka, suffix3=thy, …
distance from beginning/end	w/in1FromEdge=True, w/in2FromEdge=True, …
in word list	isProperName=True, isCommon=False, isUsername=False, …
possibly in word list	editDist1ProperName=True, editDist2ProperName=True, …
Also include features of two previous and following tokens	…
**MMB structure features**	
tf-idf over message boards	inTop10=False, inTop1%=False, …
tf-idf over user posts	InTop10=False, inTop1%=True, ...
border of paragraph likelihood	inTop5=True, inTop10%=True, ...

### Identifying names

Once feature vectors were generated for each token, a CRF name classifier was run over the tokens to estimate the marginal tag probabilities for each particular token. This corresponds to step 2 in Fig. 1. A CRF [15] is a discriminative probabilistic model that has been widely used in natural language processing in order to tag sequences. The particular name classifier that we used was trained on a 1,000-message sample from the BC corpus containing a total of 91,344 tokens, 822 proper names, and 682 usernames.

### Post-processing

After returning tag probabilities for each token, any token with a cumulative probability greater than 0.05 of being either a proper name or username was tagged as a proper name or username (whichever tag was more likely). We applied this step (step 3 in Fig. 1) in order to increase the system’s recall, without sacrificing a great deal of precision, since identifying as many names as possible is more important than preventing non-name tokens from being removed.

### Validation metrics

We validated our system using the metrics of precision, recall, F-score, and specificity. Precision was defined as the proportion of names correctly identified as names by our system over the total number of tokens our system tagged as names (3), recall as the proportion of correctly identified names out of the total number of names in the validation set (4), and F-score was the harmonic mean of precision and recall (5). Specificity was defined as the proportion of non-name tokens that were tagged as non-names by our system over the total number of non-names in the validation set (6).(3)(4)(5)(6)

## Results

In order to improve and validate our system, we created a development set with 500 messages sampled from the BC corpus (31,232 non-punctuation tokens, 483 names total) distinct from the set on which our classifier was trained, and a test set with 500 messages sampled from the arthritis corpus (28,146 non-punctuation tokens, 432 names total). Both of these sets were manually tagged by a human coder in order to evaluate the effectiveness of our system. Any token that referred to a user of the message board or anyone that they had personal contact with was tagged as a name. This may have been overly harsh since many of these tokens were acronyms or nicknames that were unlikely to identify the user.

Although the majority of these sets were tagged by a single coder (AB), a subset of 120 messages was also tagged by another coder (AC) to produce an estimate of the sole coder’s reliability. Over this subset, AB tagged 83 tokens as names and AC tagged 82 as names; their tags agreed on 81 tags (97.6% of tokens tagged as names by AB or 98.9% tagged by AC).

**Figure 2:**
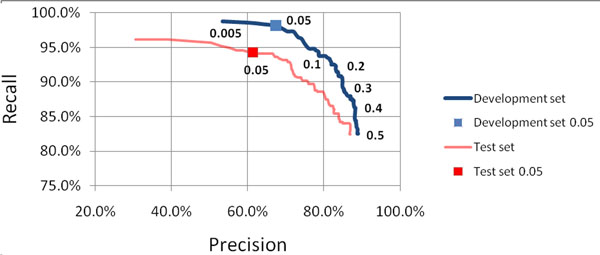
**Performance of our system over development and test sets, varying the likelihood threshold** The blue curve displays the precision and recall of our system over the development set, while varying the likelihood threshold. In this figure, the values for the likelihood threshold ranged from 0.5 to 0.005 and are displayed for major intervals on the curve. The threshold value of 0.05 was chosen, since it seemed to yield the highest recall without unnecessarily sacrificing precision over the development set. The red isolated point corresponds to the performance of our system, using the chosen threshold value of 0.05, over the arthritis corpus test set, while the blue point corresponds to its performance over the development set. The red curve corresponds to our system’s performance over the arthritis test set. Note that this curve has a similar trajectory to the performance over the BC development set and that the point of 0.05 likelihood threshold on it corresponds to a similar precision/recall trade-off as the development curve.

In order to improve our system, we experimented with several different minimum name probability thresholds for tagging a token as a name and recorded the performance of our system using each of these thresholds over the development set. Fig. 2 shows the precision-recall curve as the likelihood threshold is varied from 0.5 to 0.005. We then applied the system to the arthritis test set which resulted in a precision of 61.4% and a recall of 94.3%.

Although the F-score decreases as the name likelihood threshold decreases, the recall increases dramatically by 15.5% (82.6% to 98.1%) from the baseline of tagging tokens whose cumulative probability of being a name is greater than 50% to tagging any token whose cumulative probability exceeds 5%. For our task of de-identification, recall is much more important than precision, since the primary goal is to preserve the authors' anonymity.

Note that many tokens in the original coding of the development and test sets were unlikely to give much information as to the identity of the author. In order to accurately reflect our system’s ability to remove identifying information, a second coding of these sets was performed, where any tokens that were originally tagged as a name were tagged as “other” if they were one of: an acronym, a nickname that was obviously unrelated to the author’s username or personal name, or a substring of the username that was three or less characters long where the username was over twice that length. Our system achieved a name recall of 99.1% over the development set and 95.4% over the test set after this recoding.

The tokens where there was a discrepancy between the human coder and the system were categorized by type, as shown in Tables 3 and 4. In Table 3, “Ambig. common words” refers to tokens that were most often nicknames for users, but at times they were proper names that were misspelled as common ones (e.g., “lard” instead of “lars”). “Prop. names” refers to tokens that were clearly proper names, spelled correctly, although they may not have been in the system’s proper name list. “Abbr./Acr.” were very short nicknames no longer than three characters and often acronyms of usernames. “Misspelled usernames” were usernames that had clearly been misspelled. These were determined to be misspelled usernames by referring to the author names from the original thread. “Total N” refers to the total number of names that were not tagged as names by our system.

**Table 3:** Breakdown of the names that were not tagged as names by our system

Set	Ambig. common words	Prop. names	Abbr./ Acr.	Missp. users	Total N
DEV	50.0%	20.0%	30.0%	0.0%	10
	
TEST	48.1%	7.4%	33.3%	11.1%	27

**Table 4:** Breakdown of the tokens that were incorrectly tagged as names by our system

Set	People	Places/ Institutions	Medical	Other	Total N
DEV	35.3%	13.5%	6.3%	44.9%	207
	
TEST	32.9%	18.8%	2.9%	42.0%	200

In Table 4, “People” refers to tokens that were names of actual people, but were unrelated to any of the MMB authors (e.g., Oprah Winfrey, Tom Petty). “Places/Institutions” refers to tokens referring to a location or organization. “Medical” tokens were tokens that referred to a drug, supplement, procedure, or some other medical concept and could be useful to researchers investigating these posts. “Other” tokens could not be placed in any of the previous four categories and would probably not be very useful to researchers. Some examples of these are: “kiddo”, “june”, “april”, “morning”, “crispy”, and “sweetie”.

In order to compare our system’s performance against a currently available de-identification system, we also ran the “Deid system” [9,16] (http://www.physionet.org/physiotools/deid/) over our development and test sets. The Deid system consists of a single Perl script that relies on a combination of heuristics, regular expression, and word lists to remove identifiers. We ran the system under several conditions. The system was run first without altering any of its word lists, then by appending all the usernames on the message board to its list of ambiguous names, and finally by appending all of those usernames to the list of unambiguous names instead. The system was first judged only on its ability to identify proper names and then on its ability to identify both proper names and usernames. The system’s ability to identify usernames was poor in all cases (9.0%, 18.3%, and 67.0% recall for each of the three conditions respectively over the development set, and similarly over the test set) and the system’s precision under the final condition was prohibitively low.

We also evaluated the Stanford NER trained on a collection of US and UK newswire over both these sets. Table 5 only displays both systems’ ability to identify proper names since neither the Deid system nor the Stanford NER were designed to identify usernames, so it was unfair to evaluate their performance over these. Even adding the entire set of author names to the Deid system’s unambiguous name list resulted in a recall of only 67.0% of usernames and a drop to 11.1% precision over the development set. The poor performance of these systems over our evaluation sets does not suggest that they are bad at identifying names. It simply highlights the fact that current name identification systems must be developed for a specific domain in order to perform well in it.

**Table 5:** Performance of Deid system and Stanford named entity recognizer on development and test sets considering only proper names

		Deid - Out of the box	Deid - Ambig. name list with usernames	Deid - Unambig. name list with usernames	Stanford NER
DEV	Precision	52.1%	51.2%	11.1%	61.7%
		
	Recall	86.3%	84.2%	86.3%	81.2%
	
TEST	Precision	41.2%	40.2%	7.6%	55.4%
		
	Recall	85.1%	84.2%	85.6%	85.4%

Finally, we evaluated the performance of a freely available state-of-the-art de-identification system, MIST (The MITRE Identification Scrubber Toolkit) [17]. Like our system, MIST relies on a CRF to perform automated tagging of identifiers, and achieved the highest overall score in the de-identification task of the 2006 American Medical Informatics Association (AMIA) Challenges in Natural Language Processing for Clinical Data [13]. Both of these facts make MIST an excellent system to evaluate our system against. We trained MIST on the same set of 1,000 breast cancer MMB posts that our system was trained on and also included the dictionaries listed in Table 1 as part of its lexicon. Table 6 exhibits MIST’s performance over the BC and arthritis validation sets against the performance of our system.

**Table 6:** Performance of state-of-the-art MIST de-identification system against our system, over the development and test sets

		Development (BC)		Test (Arthritis)	
	
		Recall	Precision	Recall	Precision
**MIST**	Proper names	79.9%	80.5%	72.2%	74.9%
				
	Usernames	49.5%		34.3%	
				
	All names	73.0%		54.6%	

**Proposed system**	Proper names	98.9%	67.4%	99.0%	61.4%
				
	Usernames	94.5%		90.3%	
				
	All names	98.1%		94.3%	

In this table, recall is defined as the proportion of proper/user names that were tagged by the system with some name tag (either a proper or a user name tag, the only possible name tags) and precision was defined as the proportion of non-name tokens that the system tagged with a name tag. We used this definition of recall and precision, because the removal of identifying information is more important than the specific name tags they are replaced with.

## Discussion

Our system performs as well over MMB text as some of the other de-identification systems perform over other medical documents. In a recent challenge to remove private health information from medical discharge records [13], out of the sixteen systems evaluated, two systems exhibited an F-score of less than 78.1% and eight systems exhibited recall of less than 93.8% in identifying patient names. Given that MMB text is much noisier than discharge records, it is understandable that our system does not achieve state-of-the-art performance at de-identifying this text. However, it performs better over our MMB test sets than even the best of these systems (MIST). We believe that it is a great step forward in developing a system that can adequately de-identify medical message board text.

We chose to directly compare our system’s performance against the Deid system over the same corpus, because it was one of the few de-identification systems that were freely available. The Deid system uses a very different method of hand-tailored rules and word lists to remove identifiers. It is not surprising that this system performs poorly since it was developed for de-identifying medical records, not MMB text. The expanded word list conditions in Table 5 show either little change or no improvement in the Deid system’s performance. This is due to the fact that the word lists were expanded with author usernames, and were tokens unlikely to be labeled as proper names (Table 5 only evaluates the performance of these systems over proper names). Running the Stanford Named Entity Recognizer over a sample of 500 BC posts may be more comparable, since it also detects proper names using a CRF. Even then, its newswire-trained classifier performed with a precision of 61.7% and recall of 81.2% over just proper names in the development set (61.7% precision/69.7% recall over both proper and user names within the same sample).

Even the MIST system, which was trained on the same training set and had access to the same dictionaries as our system did not perform well. In particular, Table 6 suggests that it was unable to identify usernames well (recall of 49.5% over the development set and 34.3% over the test set), even though its training set contained explicitly marked usernames. This suggests that the default feature set that MIST uses to describe tokens is not suitable for de-identifying MMB text, although it may expressive enough to discover identifiers in more regular text, such as medical records.

The poor performance of these systems over our MMB corpus, suggests that current de-identification methods cannot readily be applied to this new text medium, and that our specialized method is useful and novel. These systems perform very well over the medium they were designed for. Neamatullah, et al. report that the Deid system was able to identify proper names in a corpus of nursing notes with 72.5% precision and 98.9% recall [9]. Finkel, Grenager, and Manning report that the Stanford NER system was able to identify person names over the Seventh Conference on Computational Natural Language Learning named entity recognition shared task with an F-score of 92.3% [6] and the MIST system achieved the best overall score in the de-identification task of the 2006 AMIA Challenges in Natural Language Processing for Clinical Data [13]. However, we show that they are unable to reliably identify names when applied to the very different medium of MMB text. We were unable to find a system specifically designed to de-identify MMB text, which is why we chose to evaluate the performance of our system against two medical record de-identification systems, Deid and MIST, and a named entity recognition system, the Stanford NER.

One of the main difficulties in identifying usernames is that many usernames are common words. Some examples of tokens ambiguous between common words and usernames that our system failed to classify were “one”, “boo”, “breezy”, “tiger”, “girl”, and “ash”. The majority of names that were missed were of this form. Another class of names that our system failed to tag was acronyms of names. However, it is difficult to imagine how a human reading the message would be able to discover the actual username based on this acronym.

The medically-related tokens that were erroneously removed by our system are of the most concern. The particular tokens that were removed from each MMB post sample are listed in Table 7. “Mastectomy”, “mris”, “dcis”, “brca1”, “brca2”, and “dieps” are all tokens that are frequently mentioned throughout the BC corpus and are for the most part unambiguously not names, so it is surprising that the classifier attributed such a great likelihood that they are names. We believe that these tokens would not have been erroneously tagged if the classifier were trained on a larger training set. “Carcinom”, “tamoxifine”, and “earlydetection” would be more difficult for our classifier to leave untagged, since they are all misspellings of actual medical terms, and these tokens are unlikely to occur with high frequency in the training corpus.

**Table 7:** Medical words incorrectly identified as names over development and test sets

DEV	TEST
brca1	doxy
brca2	doxy
carcinom [sic]	hashimoto
dcis	nurofen
dieps	sjogren
earlydetection	sjogren
mastectomy	
mastectomy	
mastectomy	
mastectomy	
mris	
oncotypedx	
tamoxifine	

The tokens removed from the test set pose a much greater concern. “Doxy” was used as an abbreviation of the pharmaceutical doxycycline, which is why it was not marked as a non-name in the post-processing step. Nicknames for drugs could pose a great problem for our system, since nicknames seem to be much more common in MMB text than in medical records, and, like “doxy”, often look very similar to usernames or nicknames of authors. “Hashimoto” and “sjogren” are difficult as well, since they are ambiguous between proper names and medical terms (Hashimoto’s disease, Sjögren’s syndrome). Within our test set, they appeared as conditions that users were discussing rather than people that they knew.

In spite of the low precision, the specificity of our system is very good, only removing about 0.7% of all non-names.

## Future work

Although our system takes a great step in de-identifying MMB text, there are several modifications that we can make in order to improve our system’s performance. First, increasing the size of the training set for the name classifier would likely improve the precision of our system by reducing the number of common words that were mistagged as names. We could also include a gazetteer of locations in order to reduce the number of mistagged places.

Second, we have not included the part of speech (POS) of the token in the feature vectors generated for tokens. Six out of the 16 systems evaluated in a 2007 discharge summary de-identification challenge [13] used POS tags to inform identification of private health information. Many statistical de-identification systems rely on this feature. As of now, we are unsure of how effective a POS tagger would be over MMB text, since these systems are often trained on text from newswires or the Wall Street Journal, which is more regular than message board text. Nevertheless, it may be worthwhile to experiment with this particular feature.

Finally, the identifiers that our system currently removes are far from full de-identification, but they are some of the most pervasive identifiers in MMB text. We intend to improve our system by specifically identifying institution names and locations as well. The removal of these terms is currently a by-product of our name classifier and we have not evaluated its performance at removing locations and institution identifiers. As investigators continue exploring MMB text to gain a greater awareness of patients’ experiences, systems such as ours will become more important than ever in protecting the privacy of the members of these communities.

## Conclusion

We have developed a system that can de-identify MMB posts by identifying and removing both proper and usernames with acceptable precision and recall. Not only is this a boon to researchers investigating these MMBs, but it also suggests that NER can be effective in even some of the noisiest forms of free text. We welcome any improvements that others can offer to our system.

## Competing interests

The authors declare that they have no competing interests.

## Authors’ contributions

AB, SH, LU, and JH all contributed to the initial design of the system. All authors participated in discussions about the interpretation of our data and results. AB and AC tagged the validation sets and evaluated their coding. AB implemented the system and drafted the manuscript. All authors contributed to revising the manuscript and provided feedback on the methods used to evaluate system performance. All authors read and approved the final manuscript.
